# The Anti-inflammatory Effect of Soluble Epoxide Hydrolase Inhibitor and 14, 15-EET in Kawasaki Disease Through PPARγ/STAT1 Signaling Pathway

**DOI:** 10.3389/fped.2020.00451

**Published:** 2020-08-12

**Authors:** Na Dai, Chunyan Yang, Qing Fan, Minmin Wang, Xiaoyue Liu, Haizhao Zhao, Cuifen Zhao

**Affiliations:** ^1^Department of Pediatrics, Qilu Hospital, Shandong University, Jinan, China; ^2^Department of Pediatrics, Jinan Maternity and Child Care Hospital, Jinan, China; ^3^Department of Pediatrics, Liaocheng People's Hospital, Liaocheng, China

**Keywords:** Kawasaki disease, 14, 15-EET, coronary injury, AUDA, PPARγ

## Abstract

Soluble epoxide hydrolase (sEH) is responsible for rapid degradation of 14, 15-EET, which is one of the isomers of EETs and plays an important role in cardiovascular diseases. In this study, we investigated the mechanism by which sEH inhibitor AUDA played an anti-inflammatory effect in HCAECs. Our results indicated that AUDA treatment promoted PPARγ expression, while knockdown of PPARγ blocked the cell growth and STAT1 expression inhibition induced by 100 μmol/L AUDA in HCAECs. AUDA also inhibited the overexpression of TNF-α, IL-1 β, and MMP-9 induced by KD sera in HCAECs. Moreover, 30 blood samples from children with Kawasaki disease (KD) were collected with 30 healthy children as the control group. QPCR and ELISA assays were used to detect the level of 14, 15-EET, TNF-α, IL-1β, and MMP-9. We found that the level of 14, 15-EET was higher in peripheral blood of children with KD compared with healthy controls (*P* < 0.05). In comparison to KD children with non-coronary artery lesion (nCAL), the level of 14, 15-EET was higher in peripheral blood of KD children with coronary artery lesion (CAL) (*P* < 0.05). Compared with healthy control group, the expression levels of TNF-α, IL-1β, and MMP-9 in patients with KD were significantly up-regulated. Compared with nCAL KD children, the expression levels of TNF-α, IL-1β, and MMP-9 in CAL children were abnormally high (*P* < 0.05). Our study indicated that AUDA played an anti-inflammatory effect in HCAECs through PPARγ/STAT1 signaling pathway, and 14, 15-EET is up-regulated in children with KD, suggesting that 14, 15-EET involved in the progression of KD.

## Introduction

Studies have shown that arachidonic acid (ARA) is converted to endogenous lipid EETs by P450 arachidonic acid cyclooxygenase (ARA cyclooxygenase), while EETs are converted to inactive DHET by sEH *in vivo* (1, 2). Cytochrome P450 2J2 (CYP2J2) is a major human ARA cyclooxygenase that produces all four EETs isomers: 5, 6-EET, 8, 9-EET, 11, 12-EET, and 14, 15-EET (3). 14, 15-EET plays an important role in cardiovascular diseases (4, 5). A number of studies have shown that 14, 15-EET promotes PDGF-induced proliferation of porcine aortic smooth muscle cells by inhibiting COX-mediated PGE2 synthesis, and has extensive cardioprotective functions, involving in a variety of cardiovascular diseases (6).

In previous studies, we found that AUDA, a sEH inhibitor, could inhibit the expression of MMP-9, IL-1β, and TNF-α *in vitro* and *in vivo*, thus protecting and repairing myocardial injury (7). In this study, we explored the specific mechanism of AUDA in the process of myocardial protection. We also collected peripheral blood samples from patients with Kawasaki disease (KD) to investigate 14, 15-EET level and its significance in coronary artery lesion (CAL). This study provides evidence for the clinical treatment of KD targeted by EETs.

## Materials and Methods

### Cell Culture and Transfection

HCAECs were obtained from the Wuhan Culture Collection. The culture and treatment with AUDA of HCAECs was performed as mentioned in our previous study (7). Cells were cultured in medium with 10% KD sera to establish KD cell model. PPARγ specific siRNA and over-expression plasmid were all purchased from Shanghai Genechem Co., Ltd. and transfected into HCAECs using Lipofectamine 2000 (Thermo Fisher Scientific, San Jose, CA, USA) following the manufacturer's instruction.

### CCK8

Cell proliferation was detected using cell counting kit-8 (CCK-8) assay (Beyotime Institute of Biotechnology, Haimen, China), which was performed according to the description in our previous study (7).

### Sample Inclusion Criteria

This study included 30 children with KD hospitalized in Qilu Hospital from June 2018 to January 2019, all of which met the fourth revised diagnostic criteria of Kawasaki Disease Research Committee of Japan (8). All children were not treated with aspirin or gamma globulin before admission. KD complicated with CAL diagnosed with *Z*-value >2.5 by the same echocardiologist (8). There was significant difference in *Z*-value between CAL and nCAL groups.

### Sample Collection

The venous blood of the normal control group and KD patients was collected 3 ml each, and centrifuged at 3,000 rpm for 10 min, and the prepared plasma was stored in a refrigerator at −80°C. Parental informed consent was obtained. The clinical data of KD and normal control were collected and compiled. The present study was approved by the ethics committee of Qilu Hospital, Shandong University and informed consents were obtained from all patients and healthy control.

### Quantitative Real-Time PCR (qPCR)

Total RNA was extracted, and then reverse transcribed to cDNA. The expression of PPARγ, STAT1, MMP-9, IL-1β, and TNF-α was detected by qPCR using SYBR Premix Ex Taq II. The relative quantification was identified by the 2^−ΔΔCt^ method after standardization to the GAPDH level. Following primers were used in this research: PPARγ-F 5′-GGCCCTGGCAAAACATTTGT-3′, PPARγ-R 5′-GATGGCCACCTCTTTGCTCT-3′, STAT1-F 5′-GGCACGCACACAAAAGTGAT-3′, STAT1-R 5′-AGAGGTCGTCTCGAGGTCAA-3′, TNF-α-F 5′-ACCGCAGTCCAGAAAGTCTC-3′, TNF-α-R 5′-TGCAGGCCTCAGGATCAAAG-3′, IL-1β-F 5′-TGGGCCTCCTCTCCTACT-3′, IL-1β-R 5′-CTTCCCCCATTCATCCCAGG-3′, MMP-9-F 5′-GACTGAGTACCTGAACCGGC-3′, MMP-9-R 5′-AGTTCCACAAAGGCATCCCAG-3′, GAPDH-F 5′-TCTCTGCTCCTCCCTGTTCT-3′, and GAPDH-R 5′-ATCCGTTCACACCGACCTTC-3′.

### ELISA

The levels of 14, 15-EET (cat. no.ab175812, Abcam), MMP-9 (cat. no.ab246539, Abcam), IL-1β (cat. no.ab100562, Abcam), and TNF-α (cat. no.ab181421, Abcam) were examined using ELISA kits.

### Statistical Analysis

Prism 5.0 (GraphPad Software, Inc., La Jolla, CA, USA) was used for data analysis. Data are expressed as the mean ± standard error. The significance of differences among several groups was determined using one-way analysis of variance and Bonferroni posttest. *P* < 0.05 was considered to indicate a statistically significant difference. All experiments in this research were performed in triplicate.

## Results

### AUDA Promotes the Proliferation of Human Coronary Arterial Endothelial Cells (HCAECs) by Up-Regulating PPARγ

**Figure 1:**
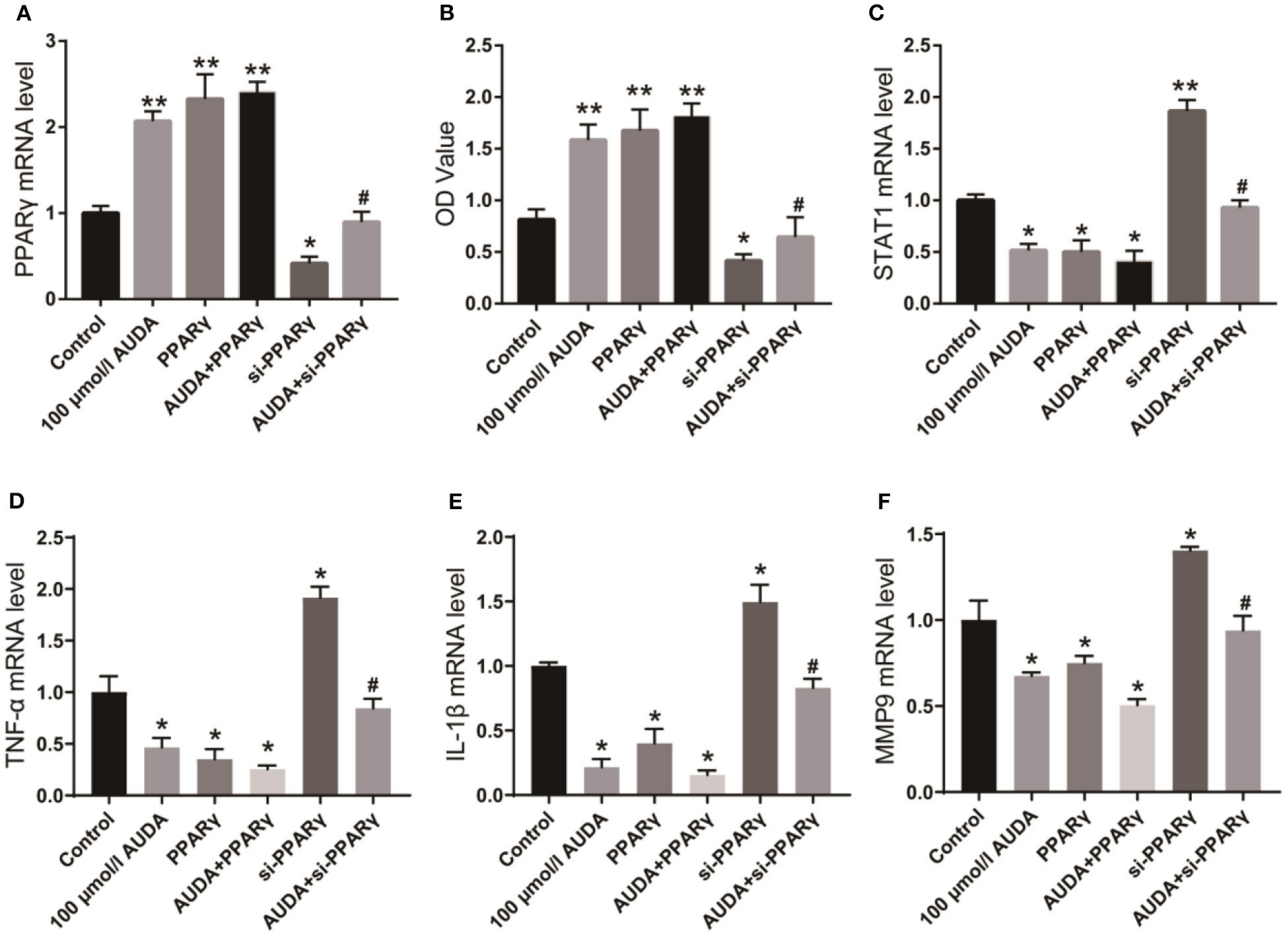
AUDA inhibited JAK/STAT1 signaling pathway by upregulating PPARγ. **(A)** The expression of PPARγ in human coronary artery endothelial cells was detected by qPCR; **(B)** The proliferation of coronary artery endothelial cells in each group was determined by CCK-8; **(C)** The expression of STAT1, a key protein in the downstream pathway of PPARγ was detected by qPCR. The mRNA levels of TNF-α **(D)**, IL-1β **(E)**, and MMP-9 **(F)** in each group were detected using qPCR. *P < 0.05, **P < 0.05 vs. control group, ^#^*P* < 0.05 vs. 100 μmol/L AUDA group.

To investigate the mechanism by which AUDA played an anti-inflammatory effect, we detected the mRNA level of PPARγ in HCAECs using qPCR. As shown in Figure 1A, the mRNA level of PPARγ increased significantly after the treatment of 100 μ mol/L AUDA, suggesting that PPARγ might be the target of AUDA in HCAECs. Then, we transfected PPARγ specific siRNA or PPARγ over-expression plasmid into HCAECs to further verify the effect of AUDA on PPARγ. From qPCR results, PPARγ expression was significantly inhibited by PPARγ siRNA and promoted by PPARγ over-expression plasmid (Figure 1A). CCK-8 assay was performed to confirm the proliferation of HCAECs. As shown in Figure 1B, the OD value of the PPARγ knockdown group (si-PPARγ) decreased significantly, while the OD value of the PPARγ over-expression group increased significantly. In addition, after the treatment of PPARγ knockdown cells with 100 μmol/L AUDA, the OD value decreased markedly compared with 100 μmol/L AUDA group (Figure 1B). Moreover, in order to investigate whether the overexpression of PPARγ formed a feedback inhibition on the proliferative effect of AUDA, we overexpressed PPARγ at the same time as AUDA treatment (AUDA+PPARγ group). The results show that the overexpression of PPARγ did not affect the effect of AUDA compared with the AUDA group (*P* < 0.05, Figure 1). These data indicated that PPARγ knockdown blocked the cell growth induced by 100 μmol/L AUDA in HCAECs.

### AUDA Inhibits JAK/STAT1 Signaling Pathway and Inflammatory Factors by Up-Regulating PPARγ

STAT1 is a critical protein of JAK/STAT1 signaling pathway, as well as a key factor in the pathogenesis and progression of inflammation (9, 10). Its downstream inflammatory factors such as TNF-α, IL-1β, and MMP-9 play crucial roles in the progression of KD (11, 12). Therefore, we investigated the effect of AUDA on STAT1 and inflammatory factors using qPCR. As shown in Figure 1C, STAT1 mRNA level decreased significantly after treatment with 100 μmol/LAUDA or overexpression of PPARγ, but increased significantly after knocking down PPARγ. After the treatment of PPARγ knockdown cells with 100 μmol/L AUDA, STAT1 expression increased to the level of control group, which was significantly higher than that of 100 μmol/L AUDA group. The expression levels of TNF-α, IL-1β, and MMP-9 were also significantly inhibited by 00 μmol/L AUDA and PPARγ, and significantly increased after PPARγ knockdown (Figures 1D,E). Moreover, PPARγ knockdown rescued the inhibition of TNF-α, IL-1β, and MMP-9 expression induced by the treatment of 100 μmol/L AUDA (Figures 1D,E). These results suggested that AUDA played an anti-inflammatory role by upregulating PPARγ and inhibiting JAK/STAT1 signaling pathway in HCAECs.

### AUDA Blocks the Inflammatory Response Induced by KD Sera in HCAECs

**Figure 2:**
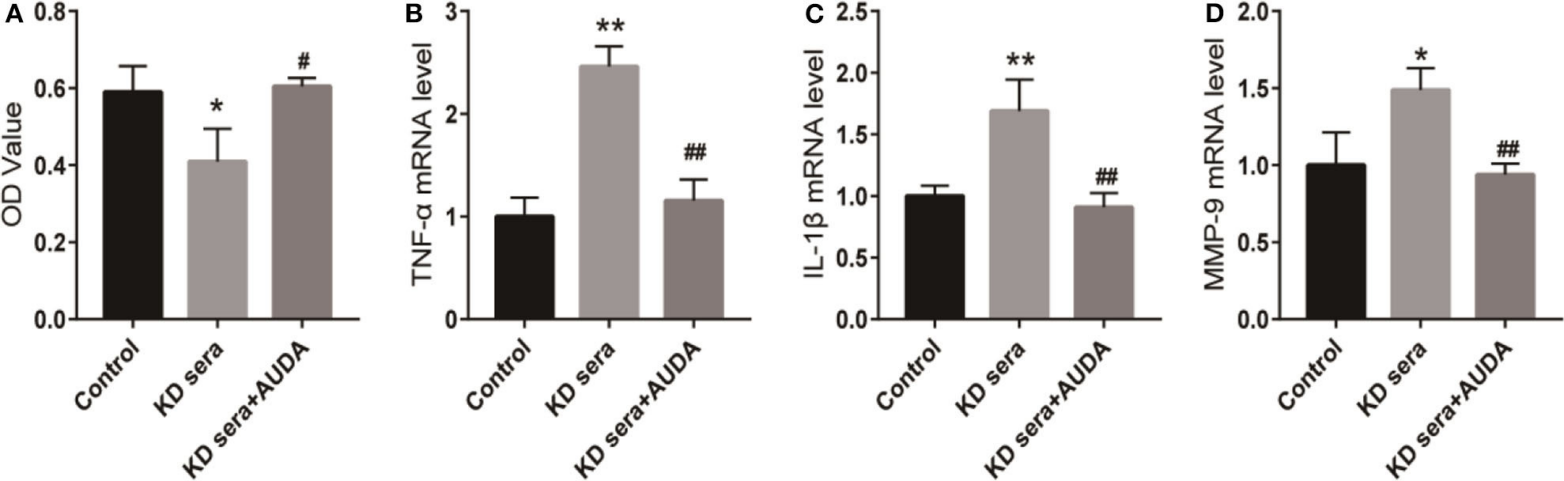
AUDA blocked the inflammatory response induced by KD sera in HCAECs. HCAECs were cultured with 10% KD sera (KD sera group) with cells in conventional culture as the control group. KD sera group cells were treated with 100 μmol/L AUDA to generate the KD sera + AUDA group. **(A)** The proliferation of HCAECs in each group was detected by CCK-8. The mRNA levels of TNF-α **(B)**, IL-1β **(C)**, and MMP-9 **(D)** in each group were detected using qPCR. *P < 0.05 vs. control; **P < 0.01 vs. control; ^#^*P* < 0.05 vs. KD sera; ^##^*P* < 0.01 vs. KD sera.

Next, we treated the 10% KD sera cultured HCAECs with 100 μmol/ L AUDA to further investigate the effect of AUDA on KD. As shown in Figure 2A, KD sera inhibited the proliferation in HCAECs compared with the control group. 100 μmol/L AUDA treatment rescued the inhibition of proliferation induced by KD sera. Moreover, the mRNA level of TNF-α, IL-1β, and MMP-9 increased in KD sera cultured HCAECs compared with the control, and decreased by the treatment of 100 μmol/L AUDA (Figures 2B–D). These results indicated that AUDA could block the inflammatory response induced by KD sera, suggesting an anti-inflammation effects of AUDA in KD sera cultured HCAECs.

### Clinical Data of Samples

A total of 30 patients with KD and 30 healthy children were enrolled in the study to detect 14, 15-EET level in peripheral blood. The average age of 30 patients with KD was 25 ± 6 m. Among them, 18 patients with CAL had an average age of 23 ± 9 m, while 12 patients without CAL had an average age of 26 ± 6 m. Compared with the normal control, the content of leukocytes, platelet, monocytes, and C-reactive protein in peripheral blood of patients with KD were significantly increased (*P* < 0.05), while the levels of leukocytes, neutrophils and C-reactive protein in peripheral blood of patients with CAL were significantly higher than those of patients without CAL (*P* < 0.05, Table 1).

**Table 1:** Blood routine test results of selected patients and normal controls.

**Groups**		**Cases (*n*)**	**Age (m)**	**Leukocyte (× 10^9^/L)**	**Platelet (× 10^9^/L)**	**Monocyte (× 10^9^/L)**	**C-reactive protein (mg/L)**
Control		30	25 ± 7	4.8 ± 0.6	220 ± 20	0.8 ± 0.3	3.5 ± 1.1
KD	nCAL	12	26 ± 6	14.5 ± 4.2^*^	320 ± 30^*^	1.7 ± 0.7^*^	49.6 ± 29.9^*^
	CAL	18	23 ± 9	19.6 ± 3.9^*^^#^	350 ± 30	2.0 ± 0.7^*^	96.9 ± 67.2^*^^#^

* *P < 0.05 vs. Control*;

# *P < 0.05 vs. nCAL*.

### 14, 15-EET Level in Peripheral Blood of Patients With KD and Its Relationship With CAL

**Figure 3:**
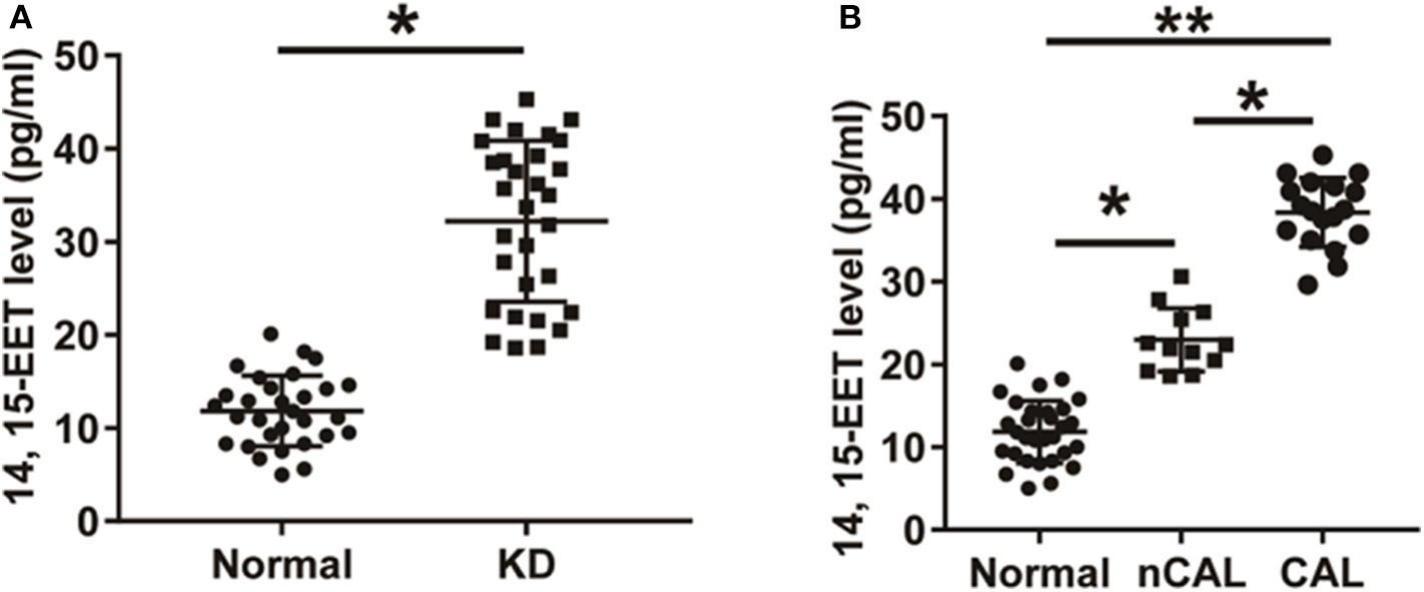
The level of 14, 15-EET in KD patients and control children. **(A)** ELISA was performed to detect 14, 15-EET level in patients with KD and normal control group. **(B)** The level of 14, 15-EET in KD patients with CAL or nCAL. *P < 0.05; **P < 0.01.

The level of 14, 15-EET in peripheral blood samples of 30 patients with KD and 30 healthy children was detected using ELISA. As shown in Figure 3A, the level of 14, 15-EET in peripheral blood of KD patients was significantly higher than that of control group, which was statistically significant (*P* < 0.01). Thirty KD patients were divided into two groups: CAL group (12 cases) and nCAL group (18 cases). The level of 14, 15-EET in the CAL group was significantly higher than that in the nCAL group via comparing 14, 15-EET level in the two groups (Figure 3B). These results suggest that 14, 15-EET is closely related to the progression of KD and plays a role in the process of CAL in patients with KD. It is a potential target for clinical intervention.

### Expression of TNF-α, IL-1β, and MMP-9 in Peripheral Blood of Patients With KD

**Figure 4:**
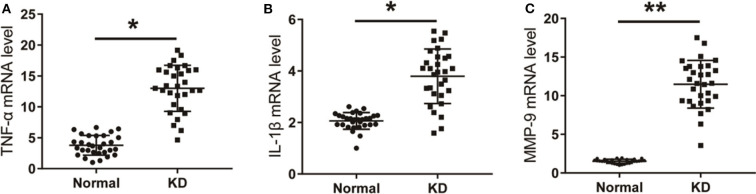
The level of inflammatory factors in KD patients. **(A)** TNF-α in KD patients and control children. **(B)** IL-1β in KD patients and control children. **(C)** MMP-9 in KD patients and control children. *P < 0.05; **P < 0.01.

**Figure 5:**
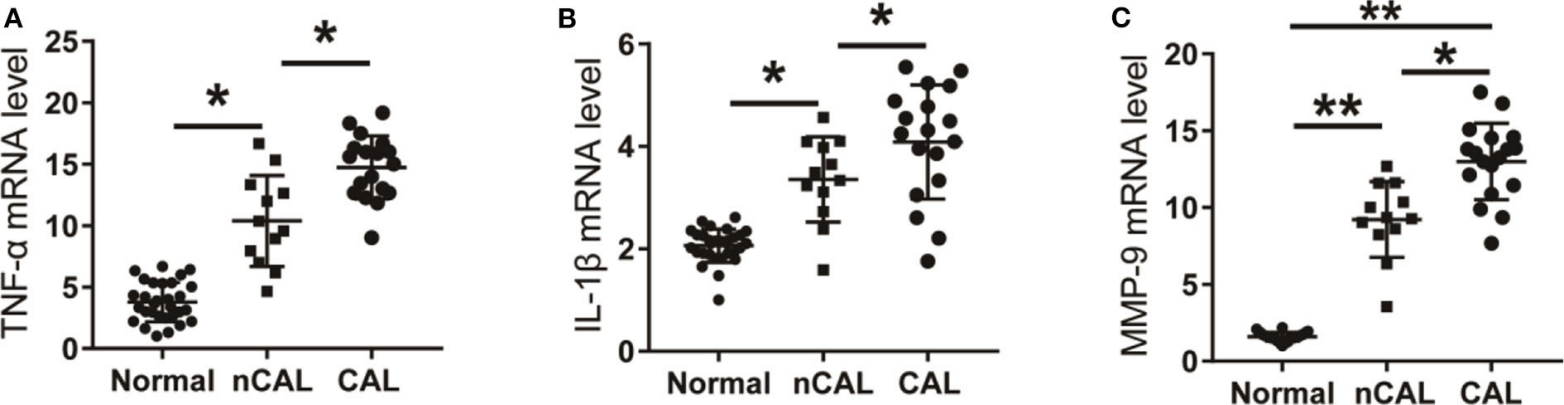
The levels of inflammatory factors in KD patients with CAL or nCAL. **(A)** TNF-α level in patients with KD. **(B)** IL-1β level in patients with KD. **(C)** MMP-9 level in patients with KD. *P < 0.05; **P < 0.01.

The mRNA levels of TNF-α, IL-1β, and MMP-9 in peripheral blood of KD patients and control children were detected using qPCR. As shown in Figure 4, the mRNA levels of TNF-α, IL-1β, and MMP-9 in peripheral blood of KD patients were significantly up-regulated compared with the control group. Results detected though ELISA showed that protein levels of TNF-α, IL-1β, and MMP-9 in peripheral blood of KD patients were also significantly up-regulated (Figure 5). These results suggest that TNF-α, IL-1β, and MMP-9 are involved in the progression of KD.

### The Relationship Between the Expression of TNF-α, IL-1β, and MMP-9 in Peripheral Blood and CAL in KD Patients

**Figure 6:**
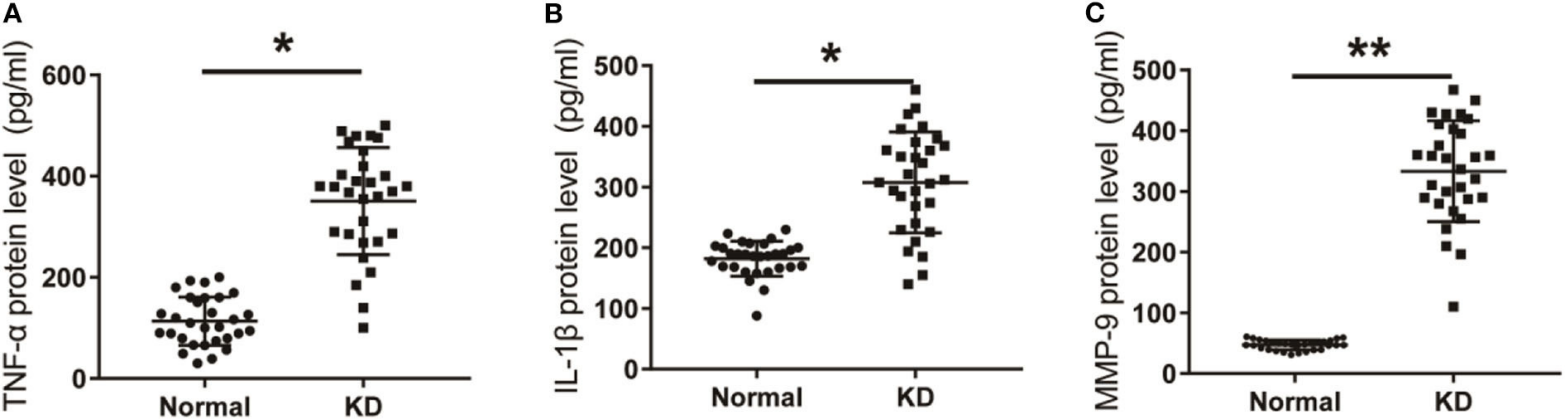
Expression of inflammatory factors in KD patients and control group. **(A)** TNF-α protein level in KD patients was detected by Elisa. **(B)** IL-1β protein level in KD patients. **(C)** MMP-9 protein level in KD patients. *P < 0.05; **P < 0.01.

**Figure 7:**
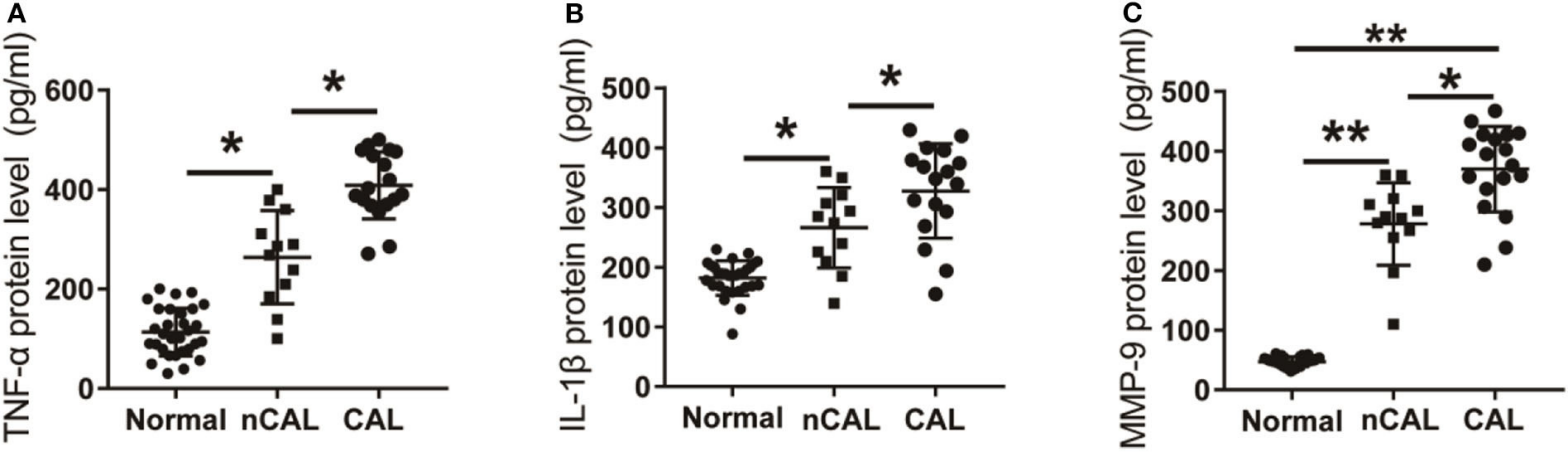
Expression of inflammatory factors in peripheral blood of KD patients and control children with CAL or nCAL. **(A)** TNF-α protein level in peripheral blood of patients with or without CAL; **(B)** IL-1β protein level in peripheral blood of patients with CAL or nCAL; **(C)** MMP-9 protein level in peripheral blood of patients with CAL or nCAL. *P < 0.05; **P < 0.01.

Further analysis of the expression levels of inflammatory factors in KD patients with CAL or nCAL showed that levels of TNF-α, IL-1β, and MMP-9 in patients with CAL were significantly higher than those in patients with nCAL (Figure 6). The protein levels of TNF-α, IL-1β, and MMP-9 in the CAL group were also significantly higher than those in the nCAL group (Figure 7). These results confirm that TNF-α, IL-1β, and MMP-9 are involved in the progress of inflammation and play roles in the development of CAL in KD patient.

## Discussion

In previous studies, we found that the proliferation, migration, adhesion, and angiogenesis were enhanced after the treatment of 100 μmol/L AUDA in HCAECs (7). PPARγ antagonist GW9662 could block the growth induced by AUDA in HCAECs, indicating that AUDA promoted the migration, adhesion, proliferation, and angiogenesis of HCAECs through the EETs-PPARγ signaling pathway (7). In this study, we knocked down/overexpressed PPARγ in HCAECs to further confirm our hypothesis. Our results proved that knockdown of PPARγ blocked the proliferation induced by AUDA, while PPARγ overexpression promoted the proliferation of HCAECs. Moreover, AUDA treatment activated STAT1 signaling pathway by upregulating PPARγ expression, suggesting an anti-inflammatory role of AUDA in HCAECs. Interestingly, AUDA could also rescue the inhibition of proliferation and increased expression of TNF-α, IL-1β, MMP-9 induced by KD sera. Thus, we hypothesized that AUDA could block the inflammatory response in KD, which needs further verification *in vitro* and *in vivo*.

EETs have significant anti-inflammatory effects and also play important roles in promoting angiogenesis and cardiovascular protection (13–15). As an important subtype of the family, 14, 15-EET plays an anti-inflammatory role in many organs (16, 17). In human endothelial cells, hypoxia can increase the level of 14, 15-EET, and CYP2C, and then inhibit the up-regulation of vascular cell adhesion molecule-1 (VCAM-1). Another study reports that 11, 12-EET and 14, 15-EET promotes the release of heparin-binding EGF-like growth factor (HB-EGF) from cell surface by up-regulating the activity of multiple MMPs family proteins (18, 19). The treatment of myocardial ischemia/reperfusion rat model with low concentration of 14, 15-EET can alleviate the arrhythmia and cardiac function changes of ischemia/reperfusion and reduce the scope of myocardial infarction. However, the role of 14, 15-EET in vascular endothelial cells remains unclear.

In the present study, the level of 14, 15-EET in peripheral blood of 30 patients with KD was detected through ELISA test. The results showed that 14, 15-EET level in peripheral blood of patients with KD was significantly higher than that of healthy controls. Further analysis showed that the level of 14, 15-EET in peripheral blood of KD patients with CAL was significantly higher than that of KD patients with nCAL. The expression of inflammatory factors TNF-α, IL-1β, and MMP-9 in peripheral blood of KD patients was significantly higher than that of normal group, and the level of inflammatory factors TNF-α, IL-1β, and MMP-9 in KD patients with CAL was significantly higher than that in patients with nCAL. The dysfunction and injury of vascular endothelial cells is the initial link of vasculitis in KD (20). Overexpression of pro-inflammatory cytokines such as TNF-α and interleukins (IL) in sera of patients with KD stimulates increased expression of vascular endothelial cell adhesion molecules including MMP-9, which in turn promotes the adhesion of neutrophils, monocytes and lymphocytes to vascular endothelium and lead to vascular endothelial cell injury (20). These results indicate that EETs are involved in anti-inflammatory regulation in the process of CAL through regulating the expression of inflammatory factors. Studies have shown that EETs are up-regulated in many diseases. In the diabetic model of mice, the increased expression of EETs significantly inhibits the apoptosis of pancreatic-β cells, and then improves the function of pancreatic-β cells (21). Some scholars have found that the level of EETs in the plasma of patients with acute heart failure increases significantly, while the level of EETs in the plasma of patients with chronic heart failure decreases. Because of the initiation of anti-inflammatory mechanism, EETs are up-regulated in the early stage of KD to play a cardioprotective role. Chronic heart failure may lead to abnormal synthesis of endogenous EETs, resulting in a decrease in EETs levels.

The high level of EETs in peripheral blood of KD patients induces cascade reactions of downstream targets and signaling pathways. Research shows that EETs are negatively correlated with MMP-9 in endothelial cells. The expression of MMP-9 in peripheral blood of KD patients was positively correlated with inflammatory factors TNF-α and L-1β. Some scholars have found that P450 cyclooxygenase or EETs can effectively inhibit homocysteine (Hcy), thereby inhibiting the binding level of transcription factor NF-kappa B to DNA, and ultimately inhibiting the level of MMP-9, through overexpression of P450 cyclooxygenase or exogenous supplementation of EETs in mouse aortic endothelial cells (MAECs) (22). 14, 15-EET has delayed cardioprotective effect, which is related to the activity of ERK and the expression of phosphorylated ERK1/ERK2 (23). In addition, EETs can also exert analgesic and anti-inflammatory effects through MAPK and cAMP/PKA signaling pathways (24–26). At present, the specific downstream targets for EETs to participate in anti-inflammation are not very clear. Several studies have shown that there are high affinity EETs binding sites in both cell membranes and cells (14). The male rats treated with exogenous EETs (100–300 mg/kg) showed a series of unique behaviors, including transient activity, exploratory behavior, and chewing. EETs binding screening of 47 potential receptors reveals that high affinity radioligands of peripheral benzodiazepine receptor (PBR), cannabinoid receptor 2 (CB2), neurokinin-2 (NK2) receptor, and dopamine D3 receptor (DRD3) are replaced by EETs with low micromolar concentration (15). In these receptors, PBR has been shown to be involved in inflammation. PBR density increases significantly after ischemic brain injury, but its specific mechanism remains unclear (27).

In conclusion, AUDA promotes the proliferation of HCAECs by upregulating the expression of PPARγ and inhibiting the JAK/STAT1 signaling pathway. 14, 15-EET is significantly up-regulated in peripheral blood of KD patients and participates in the anti-inflammatory effect of KD patients with CAL. However, the exploration of AUDA and 14, 15-EETs in this study is limited, and the related mechanisms, pharmacokinetics, and pharmacology need to be studied urgently, which will also be the focus of our future research.

## Data Availability Statement

All datasets generated for this study are included in the article/supplementary material.

## Ethics Statement

The studies involving human participants were reviewed and approved by Jinan Maternity and Child Care Hospital. Written informed consent to participate in this study was provided by the participants' legal guardian/next of kin.

## Author Contributions

ND and CY mainly performed the experiments. ND and QF analyzed the data and wrote the paper. ND, CY, MW, XL, and HZ helped with the experiments. MW helped analyzed the data. CY and CZ helped modify the paper. All authors had edited and approved the final manuscript.

## Conflict of Interest

The authors declare that the research was conducted in the absence of any commercial or financial relationships that could be construed as a potential conflict of interest.
